# The Association Between Medically Assisted Reproduction and Postpartum Depression: A Register‐Based Cohort Study

**DOI:** 10.1111/1471-0528.18127

**Published:** 2025-03-17

**Authors:** Sofie Egsgaard, Mette Bliddal, Line Riis Jølving, Xiaoqin Liu, Heidi Sonne, Trine Munk‐Olsen

**Affiliations:** ^1^ Research Unit of Child and Adolescent Psychiatry, Department of Clinical Research University of Southern Denmark Odense Denmark; ^2^ Clinical Pharmacology, Pharmacy and Environmental Medicine, Department of Public Health University of Southern Denmark Odense Denmark; ^3^ Research Unit OPEN, Department of Clinical Research University of Southern Denmark Odense Denmark; ^4^ Center for Clinical Epidemiology, Odense University Hospital, Department of Clinical Research University of Southern Denmark Odense Denmark; ^5^ NCRR‐National Centre for Register‐Based Research, Aarhus BSS Aarhus University Aarhus Denmark

**Keywords:** Infertility, medically assisted reproduction, mental health, postpartum depression, reproduction

## Abstract

**Objective:**

Undergoing medically assisted reproduction (MAR) has been linked to mixed mental health outcomes in women. We investigated the risk of postpartum depression (PPD) among mothers conceiving with MAR compared to mothers conceiving spontaneously.

**Design:**

Register‐Based Study.

**Setting:**

Denmark.

**Population:**

125 870 mothers with a PPD screening record who gave birth between 2015 and 2019.

**Methods:**

We linked mothers' PPD screening records to national health registers and defined MAR conception linking childbirths to MAR treatments.

**Main Outcome Measures:**

We defined PPD as an Edinburgh Postnatal Depression Scale score of ≥ 11. We performed logistic regression on the risk of PPD among mothers who conceived with MAR compared to spontaneous conception and further assessed variations according to duration, type, and indication for MAR.

**Results:**

The study population included 10 977 mothers with MAR conception and 114 893 with spontaneous conception, of which 767 (7%) and 8767 (8%) had PPD. We found a lower risk (adjusted risk ratio 0.87, 95% CI [0.80–0.93]) of PPD among mothers with MAR conception compared to spontaneous conception, with smaller variations according to type, duration and indication for MAR.

**Conclusions:**

We observed a lower risk of PPD among mothers with MAR conception compared to mothers with spontaneous conception. While it is unlikely that MAR itself is protective of PPD, women who seek MAR may have a strong desire and psychological preparedness for parenthood, which could explain the observed findings. Among mothers conceiving with MAR, PPD risk may vary depending on the type of MAR treatment and underlying reasons for seeking MAR.

## 1 Introduction

Infertility is a common reproductive disorder affecting 15%–20% of couples and is defined as being unable to achieve pregnancy after 1 year of trying to conceive [1]. An increasing number of couples with infertility conceive with medically assisted reproduction (MAR), including in vitro fertilisation (IVF) techniques and intrauterine inseminations (IUI), and in Denmark, one in eight children is conceived following MAR [2].

It is well‐documented that undergoing MAR is associated with a range of adverse psychological outcomes, including shame, self‐judgement and relationship dissatisfaction and some studies have also linked MAR to increased levels of distress, anxiety and depressive symptoms [3, 4, 5]. The duration of MAR may vary considerably and may, in some cases, last years. An extended treatment duration is found to additionally increase stress and anxiety levels [4].

Postpartum depression (PPD) is the most common mental disorder after childbirth [6, 7], and it has been debated whether the psychological strains from undergoing MAR increase the risk of PPD among women who conceive with MAR. Since mental health problems prior to childbirth are one of the strongest risk factors for PPD [7], women undergoing MAR could be particularly susceptible to mental health difficulties postpartum. Conversely, the desire for a child could also pose a lower risk of PPD among women who succeed in conceiving.

The existing evidence on the association between conception with MAR compared to spontaneous conception and risk of PPD or other mental health disorders during pregnancy reported mixed findings. While some studies found a higher risk of mental health challenges in the perinatal period [8, 9, 10], some reported a lower risk [11, 12, 13], and other studies reported no difference in risk [14, 15]. However, several existing studies were limited by smaller study samples of ART‐treated women, which may have insufficient power to detect associations [9, 14]. Also, MAR has often been studied as a binary exposure (yes or no), without considering the impact of treatment duration, type of treatment, and the indication—the underlying reason for seeking MAR—which potentially overlooks the complexities and nuances of undergoing MAR.

This study aimed to investigate the risk of PPD in mothers who conceived with MAR compared to mothers who conceived spontaneously. Subsequently, we investigated variations in this risk according to type, duration and indication for treatment.

## 2 Methods

We conducted a register‐based cohort study combining PPD screening records and Danish nationwide health registers in mothers of children born between 1 January 2015 and 31 December 2019.

### 2.1 Data Sources and Study Population

The study population was based on the HOPE cohort, a Danish nationwide cohort that includes PPD screening records from mothers (inclusive of birthing individuals regardless of gender identity [16]) following 170 218 childbirths [17]. Screening records were obtained from healthcare nurses' home visits on average 2 months postpartum from 67 out of 98 Danish municipalities from 2015 to 2021. The cohort has been evaluated for selection bias, showing only minor differences from the general population [17]. We restricted the study population to mothers who had given birth between 1 January 2015 and 31 December 2019, since the Danish Register of Assisted Reproductive Technology (ART Register) [18] (see below) only contains data until June 2019, which corresponds to births up to this point.

Through the unique personal identifier assigned to every individual living in Denmark [19], women were linked to the Danish nationwide health registers. The Danish Medical Birth Register (Birth Register) holds pregnancy‐ and birth‐related information of all women giving birth in Denmark, including the date of birth since 1973 [20]. The ART Register holds information on all treatments with MAR in private and public fertility clinics since 1994 [18]. In Denmark, MAR is offered free of charge to infertile couples with no shared children, single women and women in same‐sex couples for at least three cycles depending on the type of MAR treatment. The ART Register covers MAR treatments with in vitro fertilisation (IVF), intracytoplasmic sperm injection (ICSI), frozen embryo replacement (FER), oocyte donation (OD) and from 2006 also intrauterine insemination (IUI) treatments [18]. The register contains information on dates of all treatment cycles, the type of treatment used, and whether each treatment cycle resulted in pregnancy. Due to ongoing reconstruction of the ART Register, only data until June 2019 is available. The Danish National Patient Register contains information on all in‐ and outpatient hospital diagnoses and dates of contact since 1995 using the 10th International Classification of Diseases (ICD‐10) classification system [21]. The Danish National Prescription Register contains information on all redeemed prescriptions and the date of filling since 1995. Drugs in this register are classified according to the World Health Organisation (WHO) Anatomical Therapeutical Chemical (ATC) Classification [22]. Statistics Denmark holds information on sociodemographic factors including education and income level.

### 2.2 Conceiving With Medically Assisted Reproduction

We defined mothers conceiving with MAR from a treatment record in the ART Register with an achieved pregnancy that could be linked to the live birth in the Birth Register from which the PPD screening record was derived. A recorded pregnancy in the ART register had to meet the following criteria: (i) the duration between the date of MAR treatment and the date of childbirth (the plausible gestational age) was between 140 and 304 days and (ii) the plausible gestational age did not differ more than 30 days from the gestational age at birth recorded in the Birth Register [2]. Mothers who did not fulfil these criteria were defined as having conceived spontaneously. Note, since the study population was formed based on mothers who completed PPD screening records, and PPD per definition can only occur following a live birth, only successful MAR treatments resulting in a live birth were included.

We further categorised mothers who conceived with MAR by i) type of treatment used at conception (IUI, IVF/ICSI or FER/OD). We grouped IVF and ICSI together, as they typically involve extensive hormonal stimulation, and FER and OD together, due to the shared mechanism of embryo transfer, despite potential heterogeneity in infertility history or reasons for treatment; ii) treatment duration (0–3 months, 3–12 months or > 12 months) defined as the duration between first registered treatment and the treatment leading to conception. For multiparous mothers, it was defined from the first treatment date after the last childbirth; and iii) indication for MAR, that is, the underlying cause or reason for seeking MAR (female, male, joint or idiopathic infertility or single/non‐hetero women) based on registered infertility diagnoses at conception or being registered as single/non‐hetero women in MAR treatment (Table S1).

### 2.3 Postpartum Depression

The outcome of interest was PPD, which was obtained from screening records using the Edinburgh Postnatal Depression Scale (EPDS). Screening records were obtained from health nurses' home visits within 12 weeks (on average 8 weeks) postpartum. The questionnaire consists of ten questions related to sadness, self‐blame, sleep, anxiety and thoughts of self‐harm, from which women report the frequency of depressive symptoms ranging from 0 (not at all) to 3 (often or all the time) yielding a total score range between 0 and 30 points. The EPDS has been validated in Danish, in which a score of ≥ 11 indicates PPD with a sensitivity and specificity of 78.2% and 94.4% [23].

### 2.4 Covariates

Information on maternal age at childbirth and parity was obtained from the Birth Register. From Statistics Denmark, education was defined based on mothers' highest achieved education (Primary, High school or vocational, Short‐ or medium cycle and Long‐cycle or PhD), and income level was defined from the equivalized disposable family income in the year prior to childbirth and divided into income quintiles. A mother was defined as cohabiting if living with a partner in the year of childbirth. Psychiatric history was defined as any psychiatric diagnosis (ICD‐10 F00‐99) in the Patient Register or redeemed psychotropic prescriptions (ATC N05‐06) ever since 1995 and until childbirth. Further, we specifically defined the history of depression as either a depression diagnosis (ICD‐10 F32‐33) or an antidepressant prescription (ATC N06A) in the same period. A reproductive disorder (e.g., endometriosis or polycystic ovarian syndrome) was defined as any such diagnosis (ICD‐10 E28, E70‐E77, N80‐N94) [24] before childbirth, and a previous pregnancy loss was defined as a such a diagnosis (ICD‐10: O00‐O03) in the 5 years before spontaneous conception or MAR initiation.

For descriptive purposes, we further included information on country of origin (Danish or non‐Danish), multiple births, pregnancy‐ and obstetrical complications including preterm birth (birth before 37 weeks of pregnancy), caesarean section (ICD‐10: O82, O842, O843, O843D), preeclampsia/eclampsia (ICD‐10: O14, O11, O15), gestational diabetes (ICD‐10: O244), postpartum haemorrhage > 500 mL (ICD‐10: O720) and neonatal intensive care admissions (Table S1).

### 2.5 Statistical Analysis

We provided descriptive characteristics according to MAR or spontaneous conception, and for the MAR group according to type, duration and indication for MAR.

For the main analysis, we performed unadjusted and adjusted multivariable logistic regression analyses and used marginal comparisons to estimate risk ratios (RRs) and risk differences (RDs) of PPD between mothers who conceived with MAR compared to mothers who conceived spontaneously [25]. We did analyses including any MAR and by the type, duration and indication for MAR as defined above. In adjusted analyses, we included maternal age (included as restricted cubic spline), parity, education, income, psychiatric history, previous pregnancy loss, year of childbirth, cohabitation status and reproductive disorder identified based on Directed Acyclic Graphs (Figure S1). All analyses were clustered based on variance‐covariate matrix estimation, applying cluster‐robust standard errors to allow for dependence between mothers who gave birth twice or more in the cohort [26].

In secondary analyses, we stratified the overall analysis by parity of mother (primiparous or multiparous), maternal age (under or over 30 years) and psychiatric history (yes or no). As a post hoc analysis, we investigated the risk of PPD among women in MAR alone by type, duration and indication of MAR. In these, treatment duration was additionally adjusted for the indication for MAR and initial type of MAR, and the type of MAR was additionally adjusted for treatment duration and indication for MAR (Figure S2).

All analyses were conducted using R Studio version 4.2.

### 2.6 Ethics

The Danish Data Protection Agency approved this study under local registration at Aarhus University (journal number 2016–051‐000001, serial number 2304). Due to Danish legislation, register‐based studies do not require ethical approval or individual consent. The Danish Patient Safety Authority approved access to EPDS questionnaire data from health records, and the Danish Data Protection Agency granted permission to link these data with national registers at Statistics Denmark. A study protocol is pre‐registered and available at https://osf.io/qrbkm/.

## 3 Results

The study population consisted of 125 870 mothers (111 462 unique), including 10 977 (8.7%) who conceived with MAR and 114 893 who conceived spontaneously, of which 767 (7%) and 8767 (8%) had PPD (Table 1). Mothers who conceived with MAR were on average older, primiparous, had a psychiatric history, high education and income levels, pregnancy‐ and obstetrical complications, and reproductive disorders, but were less likely to be cohabiting with a partner compared to women who conceived spontaneously. Among mothers who conceived with MAR, most conceived with IVF/ICSI (39%), had treatment duration between 0 and 3 months (44%), and had female (26%) or male (25%) infertility as indications for MAR (Table 1).

**TABLE 1:** Characteristics of the study population including mothers in the HOPE cohort giving birth in Denmark between 1 January 2015, and 31 December 2019.

	Conception with MAR	Spontaneous conception
	*n* = 10 977	*n* = 114 893
Age, median (IQR)	33 (30–37)	30 (27–33)
Cohabitation status (%)		
Cohabiting	9543 (87)	106 981 (93)
Non‐cohabiting	1427 (13)	7791 (7)
Educational level (%)		
Primary	534 (5)	12 264 (11)
High school or vocational	2888 (26)	35 330 (31)
Short‐ or medium cycle	4262 (39)	40 638 (35)
Long‐cycle or PhD	3252 (30)	25 877 (23)
Income quintile (%)		
Lowest	1112 (10)	23 927 (21)
Second	1832 (17)	23 205 (20)
Third	2149 (20)	22 888 (20)
Fourth	2495 (23)	22 543 (20)
Highest	3341 (30)	21 697 (19)
Country of origin (%)		
Denmark	9809 (89)	98 761 (86)
Foreign	1161 (11)	16 011 (14)
Psychiatric history (%)	3750 (34)	34 176 (30)
Psychiatric history of depression (%)	2334 (21)	21 887 (19)
Reproductive disorder (%)	1797 (16)	10 422 (9)
Previous pregnancy loss (%)	1028 (9)	14 498 (13)
Year of childbirth (%)		
2015–2017	5930 (54)	65 142 (57)
2018–2019	5047 (46)	49 751 (43)
Parity (%)		
Primiparous	7552 (69)	59 594 (52)
Multiparous	3364 (31)	54 175 (47)
Preeclampsia/Eclampsia (%)	557 (5)	3652 (3)
Gestational diabetes (%)	670 (6)	4972 (4)
Postpartum haemorrhage (%)	1352 (12)	7926 (7)
Multiple births (%)	456 (4)	1109 (1)
Preterm birth (%)	712 (6)	4474 (4)
Caesarean section (%)	2267 (21)	14 148 (12)
Neonatal intensive care admission (%)	854 (8)	6552 (6)
Postpartum depression (EPDS ≥ 11) (%)	767 (7)	8767 (8)
EDPS score (Median (IQR))	4 (2–7)	4 (2–7)
Type of MAR used at conception (%)		
IVF/ICSI	4233 (39)	
FER/OD	2883 (26)	
IUI	3861 (35)	
Duration of MAR (%)		
0–3 months	4871 (44)	
3–12 months	3491 (32)	
> 12 months	2615 (24)	
Indication for MAR (%)		
Female infertility	2874 (26)	
Male infertility	2744 (25)	
Joint infertility	1555 (14)	
Idiopathic infertility	2038 (19)	
Single/non‐hetero women	1766 (16)	

*Note:* Some cells do not sum up to the total population due to missing values (all proportions of missing were < 2%).

Abbreviations: EPDS, Edinburgh Postnatal Depression Scale; FER, Frozen embryo replacement; ICSI, Intracytoplasmic sperm injection; IQR, Interquartile range; IUI, intrauterine insemination; IVF, In vitro fertilisation; MAR, Medically assisted reproduction; OD, Oocyte donation.

**FIGURE 1:**
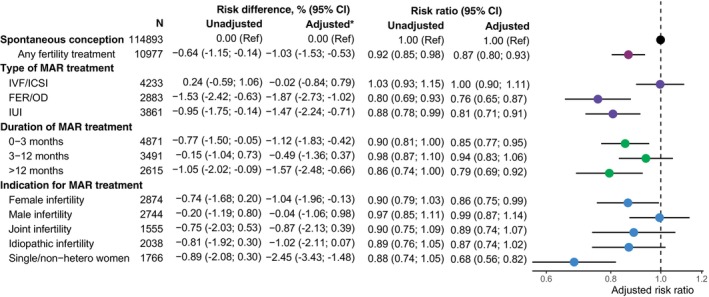
Unadjusted and adjusted risk ratio of PPD in mothers who conceived with MAR overall and by type, duration, and indication for MAR compared to mothers who conceived spontaneously. Adjusted analyses include age, parity, cohabitation, education, income, reproductive disorders, previous pregnancy loss, psychiatric history, and year of childbirth. 2524 (2,0%) with missing information excluded in adjusted analyses. Abbreviations: FER: Frozen embryo replacement; IVF: In vitro fertilization; ICSI: Intracytoplasmic sperm injection; IUI: Intrauterine insemination; MAR: Medically assisted reproduction; OD: Oocyte donation.

In the main analysis, the adjusted RR and RD of PPD were 0.87 (95% CI 0.80–0.93) and −1.03% (95% CI −1.53%; −0.53%) among mothers who conceived with any MAR compared to mothers who conceived spontaneously (Figure 1). When stratifying for type, duration and indication for MAR, the adjusted RR and RD were 1.00 (95% CI 0.90–1.11) and −0.02% (95% CI −0.84%; 0.79%) for women treated with IVF/ICSI and lowest among mothers in MAR who were single/non‐hetero (RR 0.68, 95% CI 0.56–0.82 and RD −2.45%, 95% CI −3.43%; −1.48%) (Figure 1).

**FIGURE 2:**
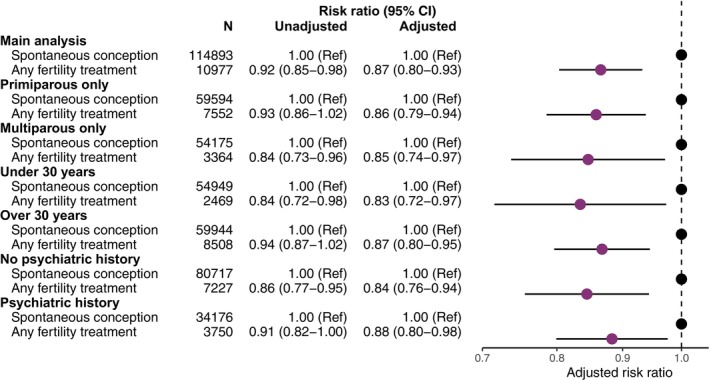
Unadjusted and adjusted risk ratio of PPD in mothers who conceived with MAR compared to mothers who conceived spontaneously stratified by parity, age, and psychiatric history. Adjusted analyses include age, parity, cohabitation status, education, income, reproductive disorders, previous pregnancy loss, psychiatric history, and year of childbirth. Abbreviations: MAR: Medically assisted reproduction.

Analyses stratified by age, parity and psychiatric history yielded very similar results to the main analysis, with adjusted RRs ranging between 0.83 (95% CI 0.72–0.97) for mothers under 30 years and 0.88 (95% CI 0.80–0.98) for mothers with a psychiatric history (Figure 2, Table S3).

**FIGURE 3:**
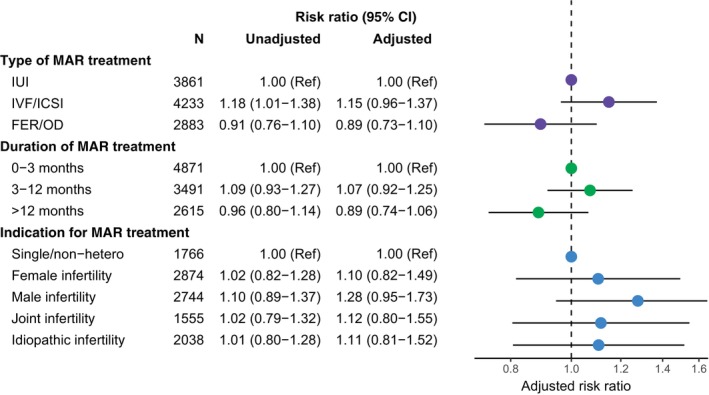
Unadjusted and adjusted risk ratio of PPD among mothers who conceived with MAR according to the type of MAR at conception, duration of MAR, and indication for MAR. Adjusted analyses include age, parity, cohabitation status, education, income, reproductive disorders, previous pregnancy loss, psychiatric history, and year of childbirth. The type of MAR treatment used at conception was further adjusted for the indication for MAR and treatment duration. Duration of MAR was further adjusted for the indication for MAR and initial type of MAR treatment. 144 (1.3%) with missing information excluded in adjusted analyses. Abbreviations: FER: Frozen embryo replacement; IVF: In vitro fertilization; ICSI: Intracytoplasmic sperm injection; IUI: intrauterine insemination: MAR: Medically assisted reproduction; OD: Oocyte donation.

Among mothers who conceived with MAR, the adjusted RR was marginally elevated among mothers treated with IVF/ICSI (1.15, 95% CI 0.96–1.37) compared to women treated with IUI (Figure 3, Table S3). Compared to a treatment duration of 0–3 months, the adjusted RR was slightly elevated among women with a duration of 3–12 months (1.07, 95% CI 0.92–1.25) but lower among durations > 12 months (0.89, 95% CI 0.74–1.06). The RR of PPD was highest in women with a partner with male infertility (1.28, 95% CI 0.95–1.73) and slightly elevated for female, joint and idiopathic causes of infertility compared to single/non‐hetero women in MAR (Figure 3).

## 4 Discussion

### 4.1 Main Findings

In this study of 125 870 mothers, we found a lower risk of PPD among mothers who had conceived with MAR compared to mothers who conceived spontaneously. Risk ratios were consistently lower when categorising by type, duration and indication for MAR, except for IVF/ICSI treatment, where the adjusted RR was unchanged. Stratified analyses on age, parity and psychiatric history did not change the findings. However, when restricting our analyses to the MAR group only, our findings indicated slightly elevated risks among women who had conceived from IVF/ICSI compared to IUI and among those in treatment with indications of infertility compared to single/non‐hetero women in treatment.

### 4.2 Strengths and Limitations

Strengths of this study included the linkage between the HOPE cohort, a large representative sample of mothers, and register‐based information on MAR treatment, pregnancy, birth and sociodemographic information, ensuring negligible selection bias and high information validity. While register‐based studies are often limited by only having hospital‐registered diagnoses or antidepressant prescription medication to identify or approximate PPD, we were able to include symptom scores using the EPDS, which captures a wider range of PPD cases. Though it should also be recognised that despite being validated against ICD‐10 depression diagnosis [23], misclassification of PPD status is likely, which could result in non‐differential misclassification and bias towards the null. Further, while dichotomising EPDS improves clinical interpretability, it does not capture the nuances in EPDS scores (Figure S3). Additionally, several limitations should be addressed:

Most importantly, we were not able to control for all possible confounders, such as pregnancy intention or desire for motherhood, or rule out the presence of ‘healthy‐user bias’, which could have biased our association downwards (see *interpretation* section for elaboration).

Secondly, we defined a birth conceived with MAR based on the duration between a pregnancy in the ART register and childbirth and defined all other pregnancies as conceived spontaneously, ignoring previous MAR. Thus, women who underwent MAR but conceived spontaneously would be categorised as conceiving spontaneously, despite potential infertility issues, which would have biased the observed association towards the null.

Third, we classified MAR by type, duration and indication for treatment to detect possible nuances in PPD risk, recognising that they are likely to be intertwined. Further, we defined the type of MAR based on the treatment used at conception and did not take any previous MAR treatments into account. We defined duration as the difference between the first and last treatment date, although it is possible that MAR treatment could have been paused in this time span. Also, we used infertility diagnoses in the ART register to classify indications for treatment, although the validity of this is unknown, and we classified single/non‐hereto women as such regardless of infertility diagnoses, which could have led to misclassification.

Fourth, this study included only mothers with live births, as PPD, by definition, only relates to women who have given birth to a liveborn child. Thus, it should be acknowledged that this does not reflect the mental health among women with unsuccessful MAR treatment. Lastly, the study was conducted in a Danish setting where women have free access to MAR and in an ethnically homogeneous population. Thus, our findings may not be generalisable to settings with different health systems and regulations regarding MAR or more ethnically diverse populations.

### 4.3 Interpretation

Previous studies found mixed associations between mothers who conceived with MAR compared to spontaneous conception and the risk of PPD [8, 10, 11, 15]. Some studies found a lower risk of PPD and lower levels of depression and anxiety during pregnancy among women who conceived with MAR [11, 12], which is in line with the findings from our study. Contrary to our findings, one study also found an increased risk of initiating antidepressants in the first year postpartum among mothers who conceived with MAR compared to spontaneous conception [8]. While some previous studies were limited by small populations of women conceiving with MAR, we were able to describe the risk of PPD in a large population of more than 10,000 women who conceived with MAR. Further, we were able to describe these risks according to duration, type and indication for seeking MAR, enabling us to identify possible variabilities within MAR treatment.

Our finding of an overall lower risk of PPD among women who conceived with MAR compared to women who conceived spontaneously contradicts our initial hypothesis that a negative impact from MAR on mental health could increase susceptibility to PPD. While undergoing MAR may, in fact, not increase the risk of mothers developing PPD, we argue that it is intuitively unlikely that it is causally *protective* of PPD. Based on the E‐value [27], fully explaining the observed association would require a confounder (or combination of confounders) with a risk ratio of at least 1.56 (Table S4). We argue that the following explanations may account for unobserved confounding:

First, women in MAR have actively sought and planned their pregnancies, whereas it is uncertain to which extent women who conceived spontaneously had planned their pregnancies or not. Unplanned pregnancies have been linked to an increased risk of PPD [28], possibly related to increased psychological stress from interruptions in education, career or other life aspirations [29]. If women who conceived with MAR had a higher psychological preparedness or desire for motherhood, this may explain our findings of a lower risk of PPD in this group.

Second, studies have suggested that women seek MAR in periods of stable mental health [30]. Even though a higher proportion of women in MAR had a psychiatric history, it is possible that they sought treatment in a period of stable “recent” mental health compared to women who conceived spontaneously. However, adjusting for mental health in various ways, including only 5 years before childbirth, showed similar results (Table S2).

Third, it is also likely that the observed association reflects a “healthy user bias”‐phenomenon, meaning that women who seek and conceive through MAR may have a generally greater health awareness, stronger partner relationships and better support systems, which could not be accounted for in the analyses. Women who conceived with MAR were sociodemographically advantaged with higher education and income levels than women who conceived spontaneously. Despite free access to MAR in public hospitals in Denmark, it has been documented that the uptake of both private and public MAR has a strong social gradient [31]. Although we included sociodemographic characteristics such as education and income level in the analyses, and our sensitivity analyses yielded similar estimates (Table S2), it remains highly likely that healthy user bias influences the observed association.

Among the subgroup of mothers in MAR, we found a slightly elevated risk (RR 1.15, 95% CI 0.96–1.37) of PPD among women treated with IVF/ICSI compared to less extensive treatments, suggesting that a greater treatment burden prior to conceiving may affect mental health in motherhood. While a longer treatment duration could hypothesise an increased risk of PPD, we observed only a marginally elevated risk among mothers with a duration of 3–12 months and lower risk for durations > 12 months. However, is it possible that the lower risk in women with > 12 months treatment duration may reflect a “healthy survivor” effect, where only those with the mental resilience, support and resources continued treatment, while others may have stopped and did not conceive. We found that the risk of PPD was lowest among single/non‐hetero women, who did not necessarily suffer from infertility, compared to couples who had indications of infertility. This may suggest that stressors of infertility and the mental impact of being unable to conceive spontaneously could be associated with an elevated risk of PPD, though estimates were uncertain.

## 5 Conclusions

We found an overall lower risk of PPD among mothers who conceived with MAR compared to mothers who conceived spontaneously. However, when stratifying by type of MAR treatment, women who conceived with IVF/ICSI treatment had no difference in risk from spontaneous conception. While it is unlikely that undergoing MAR itself reduces the risk of PPD, we speculate that women who conceived with MAR represent a group with a high desire and psychological preparedness for parenthood who sought pregnancy in a period of stable mental health. We observed smaller variations in PPD risk among mothers conceiving with MAR according to the type of MAR treatment, as well as indication for seeking MAR, implying that more extensive types of MAR treatment or having struggled with infertility may impact the risk of PPD in new mothers. Despite the overall observed lower risk, it is important to recognise that women who have conceived with MAR can still develop PPD, and they remain an important group to address in postpartum mental health care. Future studies could disentangle the influence of psychological resilience and mental health from undergoing MAR and transitioning to the postpartum period and thus improve the understanding of mental health in mothers who have conceived with MAR.

## Author Contributions

S.E., M.B., L.R.J., X.L., H.S. and T.M‐.O. took part in study design, conceptualization, interpretation, reviewing and editing the manuscript. S.E. and X.L. had access to the data, and S.E. was responsible for data analysis and writing original drafts of the manuscript.

## Disclosure

T.M‐.O. has received a speaker honorarium from Lundbeck A/S. Remaining authors: None.

## Ethics Statement

The Danish Data Protection Agency approved this study under local registration at Aarhus University (journal number 2016–051‐000001, serial number 2304). Due to Danish legislation, register‐based studies do not require ethical approval or individual consent. The Danish Patient Safety Authority approved access to EPDS questionnaire data from health records, and the Danish Data Protection Agency granted permission to link these data with national registers at Statistics Denmark.

## Conflicts of Interest

The authors declare no conflicts of interest.

## Supporting information


Data S1.

## Data Availability

The current study uses individual‐level register data. This is confidential and cannot be shared according to Danish regulations. Information on application for data access is available from https://sundhedsdatastyrelsen.dk/da/forskerservice/ansog‐om‐data.
