# Supplementary material for: The Association Between Medically Assisted Reproduction and Postpartum Depression: A Register‐Based Cohort Study
Source: BJOG. 2025 Mar 17;132(7):991–9. doi: 10.1111/1471-0528.18127 (PMC12051253; doi:10.1111/1471-0528.18127)
Supplement: Supplementary file 1 — Data S1. [file BJO-132-991-s001.docx]

**Supplement**

**Tables**

**Table S1: Definition of exposure and covariates defined from the Danish National Register of Assisted Reproductive Technology, the Danish Medical Birth Register and the National Patient Register**

| The Danish National Register of Assisted Reproductive Technology | | | |
| --- | --- | --- | --- |
| Variable | **Definition** *(Variables)^a^* | | **Comment** |
| Childbirth following MAR | Record of achieved pregnancy (*v_gravid)* in the ART register which could be linked to a livebirth in the Birth Register which fulfilled that: (i) the duration between ART treatment date (*d_startdato)* and date of childbirth (the assumed duration of pregnancy) did not exceed 308 days and (ii) if this duration did not differ more than 30 days from registered gestational age at birth. | | In case of several eligible treatment dates in which the pregnancy could had occurred, the last one was chosen. For childbirths with missing information on gestational age (~1%) we considered the birth obtained from ART-treatment if the assumed pregnancy duration was between 140 and 308 days |
| Type of MAR | The type of treatment that was registered *(c_behandling)* at the treatment defined as leading to childbirth | | Type of MAR was defined regardless of previous types of MAR used. |
| Duration of MAR | The numerical difference between the first registered treatment date *(d_startdato )* in the ART register that had not been cancelled *(b_behandling_afbrudt)* and date of treatment leading to the included pregnancy. For multiparous mothers defined as first registered treatment date after the preceding childbirth. | | Duration was defined regardless of type of MARs used over time. |
| Indication for MAR | Indication for MAR was classified based on the primary female and male ICD-10 diagnosis codes *(c_kvinde primaer, c_mand_primaer),* indication of idiopathic cause (*b_idiopatisk),* and cohabitation *(v_samlivs_status)* that was registered in the ART register at the treatment defined as leading to childbirth. | | The definitions listed are hierarchical (i.e. in case of fulfilling more than one definition, the first listed definition that is fulfilled defines the infertility cause) |
|  | **Single/non-hetero:** Cohabitation status indicating MAR of single women or women with female partner (Danish: “Enlig” or “Kvindelig partner”) | |  |
|  | **Both male and female infertility:** Any registered male diagnosis (excluding EZDH01) AND any registered female diagnosis (excluding DN974) | |  |
|  | **Female infertility:** Any registered female diagnosis excluding DN974 | |  |
|  | **Male infertility:** Any registered male diagnosis excluding EZDH01 | |  |
|  | **Idiopathic:** Registered idiopathic cause of infertility OR female and male diagnosis with DN974 and EZDH01 | |  |
| ^a^Parentheses in this column indicate variables used to define the variable from the table *t_grund* in the ART Register from 2006-2019 | | | |
| The Medical Birth Register and The National Patient Register | | | |
| Variable | **ICD-10 Code** | **Register ascertainment window (relative to date of childbirth)** | **Comment** |
| Preeclampsia/eclampsia | O14, O11, O15 | [- GA at delivery: +7] | Diagnosis code mother |
| Gestational diabetes | O244 | [- GA at delivery: +7] | Diagnosis code mother |
| Cesaeran section | O82, O842, O843, O843D | [-7 : +7] | Diagnosis code mother |
| Postpartum hemorrage | O720 (+) VPH* | [-7 : +7] | Diagnosis code mother. Supplemented w. supplementary code (+) VPH500 |
| Neonatal care admission |  | [0 : +1] | Diagnosis code child. Supplemented w. hospital unit specialty 080 ("Pædiatri") (English: Pediatric) |
| Reproductive disorder | E28, E70-E77, N80-N94 | [Ever : 0] | Diagnosis code mother |
| Previous pregnancy loss | O00-O03 | [-5 years;start*] | * Start representing either start of pregnancy or start of MAR initiation. |
| The National Patient Register and the National Prescription Register | | | |
| Variable | **ICD-10 Code and/or ATC code** | **Register ascertainment window (relative to date of childbirth)** | **Comment** |
| Psychiatric history | ICD-10 F00-99 or  ATC N05-6 | [Ever : 0] | Diagnosis code mother |
| Psychiatric history of depression | ICD-10 F32-33 or  ATC N06A | [Ever : 0] | Diagnosis code mother |

| **Sensitivity analysis** | **Exposure** | **n** | **Risk ratio (95% CI)** |
| --- | --- | --- | --- |
| **Income** |  |  |  |
| Income as in main analysis | Spontaneous conception | 114893 | 1.00 (Ref) |
|  | MAR treatment | 10977 | 0.87 (0.80-0.93) |
| Income adjusted in deciles | Spontaneous conception | 114893 | 1.00 (Ref) |
|  | MAR treatment | 10977 | 0.87 (0.81-0.94) |
| Income adjusted as a spline with five knots | Spontaneous conception | 114893 | 1.00 (Ref) |
|  | MAR treatment | 10977 | 0.86 (0.80-0.93) |
| Family income adjusted in deciles | Spontaneous conception | 114893 | 1.00 (Ref) |
|  | MAR treatment | 10977 | 0.86 (0.80-0.92) |
| Family income adjusted as a spline with five knots | Spontaneous conception | 114893 | 1.00 (Ref) |
|  | MAR treatment | 10977 | 0.86 (0.79-0.92) |
| **Psychiatric history** |  |  | 1.00 (Ref) |
| Previous psychiatry as in main analysis | Spontaneous conception | 114893 | 0.87 (0.80-0.93) |
|  | MAR treatment | 10977 | 1.00 (Ref) |
| Previous psychiatry only last five years | Spontaneous conception | 114893 | 0.89 (0.82-0.95) |
|  | MAR treatment | 10977 | 1.00 (Ref) |
| Previous psychiatry prior to conception/pregnancy | Spontaneous conception | 114893 | 0.87 (0.81-0.94) |
|  | MAR treatment | 10977 | 1.00 (Ref) |
| Adjusting for previous depression only | Spontaneous conception | 114893 | 0.87 (0.81-0.94) |
|  | MAR treatment | 10977 | 1.00 (Ref) |
| Adjusting for previous depression only in last five  years | Spontaneous conception | 114893 | 0.88 (0.82-0.95) |
|  | MAR treatment | 10977 | 1.00 (Ref) |
| Adjusting for previous depression prior to  conception/pregnancy | Spontaneous conception | 114893 | 0.88 (0.81-0.94) |
|  | MAR treatment | 10977 | 1.00 (Ref) |
| **Definition of MAR** |  |  |  |
| Defining all women with a record of MAR prior to  childbirth as conceiving with MAR. | Spontaneous conception | 109274 | 1.00 (Ref) |
|  | MAR treatment | 16596 | 0.92 (0.87-0.98) |
| *All analysis adjusted for age, parity, cohabitation status, education, income, reproductive disorders, psychiatric history, and year of childbirth* | | | |

**Table S2: Sensitivity analysis adjusting for income and depression history in various ways**

**Table S3: Risk differences in stratified analysis and anslysis of IVF only**

|  |  |  | **Risk difference, % (95% CI)** | |
| --- | --- | --- | --- | --- |
|  | **Exposure** | **n** | **Unadjusted** | **Adjusted** |
| **Stratified analysis** |  |  |  |  |
| Primiparous only | Spontaneous conception | 114893 | 0.00 (Ref) | 0.00 (Ref) |
|  | MAR treatment | 10977 | -0.52 (-1.15; 0.11) | -1.13 (-1.74; -0.51) |
| Multiparous only | Spontaneous conception | 114893 | 0.00 (Ref) | 0.00 (Ref) |
|  | MAR treatment | 10977 | -1.17 (-2.02; -0.33) | -1.13 (-1.99; -0.27) |
| Under 30 years | Spontaneous conception | 114893 | 0.00 (Ref) | 0.00 (Ref) |
|  | MAR treatment | 10977 | -1.24 (-2.24; -0.25) | -1.27 (-2.26; -0.28) |
| Over 30 years | Spontaneous conception | 114893 | 0.00 (Ref) | 0.00 (Ref) |
|  | MAR treatment | 10977 | -0.44 (-1.02; 0.15) | -1.01 (-1.59; -0.43) |
| No psychiatric history | Spontaneous conception | 114893 | 0.00 (Ref) | 0.00 (Ref) |
|  | MAR treatment | 10977 | -0.82 (-1.34; -0.29) | -0.88 (-1.42; -0.34) |
| Psychiatric history | Spontaneous conception | 114893 | 0.00 (Ref) | 0.00 (Ref) |
|  | MAR treatment | 10977 | -1.16 (-2.22; -0.09) | -1.44 (-2.53; -0.34) |
| **MAR treatment only** |  |  |  |  |
| Type of MAR treatment | IUI | 3861 | 0.00 (Ref) | 0.00 (Ref) |
|  | IVF/ICSI | 4233 | 1.18 (0.05 - 2.32) | 1.01 (-0.27 - 2.29) |
|  | FER/OD | 2883 | -0.58 (-1.76 - 0.60) | -0.73 (-2.06 - 0.60) |
| Duration of MAR treatment | 0-3 months | 4871 | 0.00 (Ref) | 0.00 (Ref) |
|  | 3-12 months | 3491 | 0.62 (-0.51 - 1.74) | 0.50 (-0.65 - 1.65) |
|  | >12 months | 2615 | -0.28 (-1.46 - 0.90) | -0.81 (-2.01 - 0.40) |
| Indication for MAR treatment | Single/non-hetero women | 1766 | 0.00 (Ref) | 0.00 (Ref) |
|  | Female infertility | 2874 | 0.15 (-1.35 - 1.65) | 0.15 (-1.35 - 1.65) |
|  | Male infertility | 2744 | 0.70 (-0.84 - 2.23) | 0.70 (-0.84 - 2.23) |
|  | Joint infertility | 1555 | 0.14 (-1.59 - 1.87) | 0.14 (-1.59 - 1.87) |
|  | Idiopathic infertility | 2038 | 0.08 (-1.53 - 1.69) | 0.08 (-1.53 - 1.69) |
| *All analysis adjusted for age, parity, cohabitation status, education, income, reproductive disorders, psychiatric history, previous pregnancy loss and year of childbirth* | | | |  |

**Table S4:**  Calculation of E-value

| E-value formula | $E_{value}=RR+\sqrt{RR*\left( RR-1 \right)}$ |
| --- | --- |
| E-value of main estimate | $E_{value}=\frac{1}{0.87}+\sqrt{\frac{1}{0.87}*\left( \frac{1}{0.87}-1 \right)}=1.56$ |

**Figures**

**
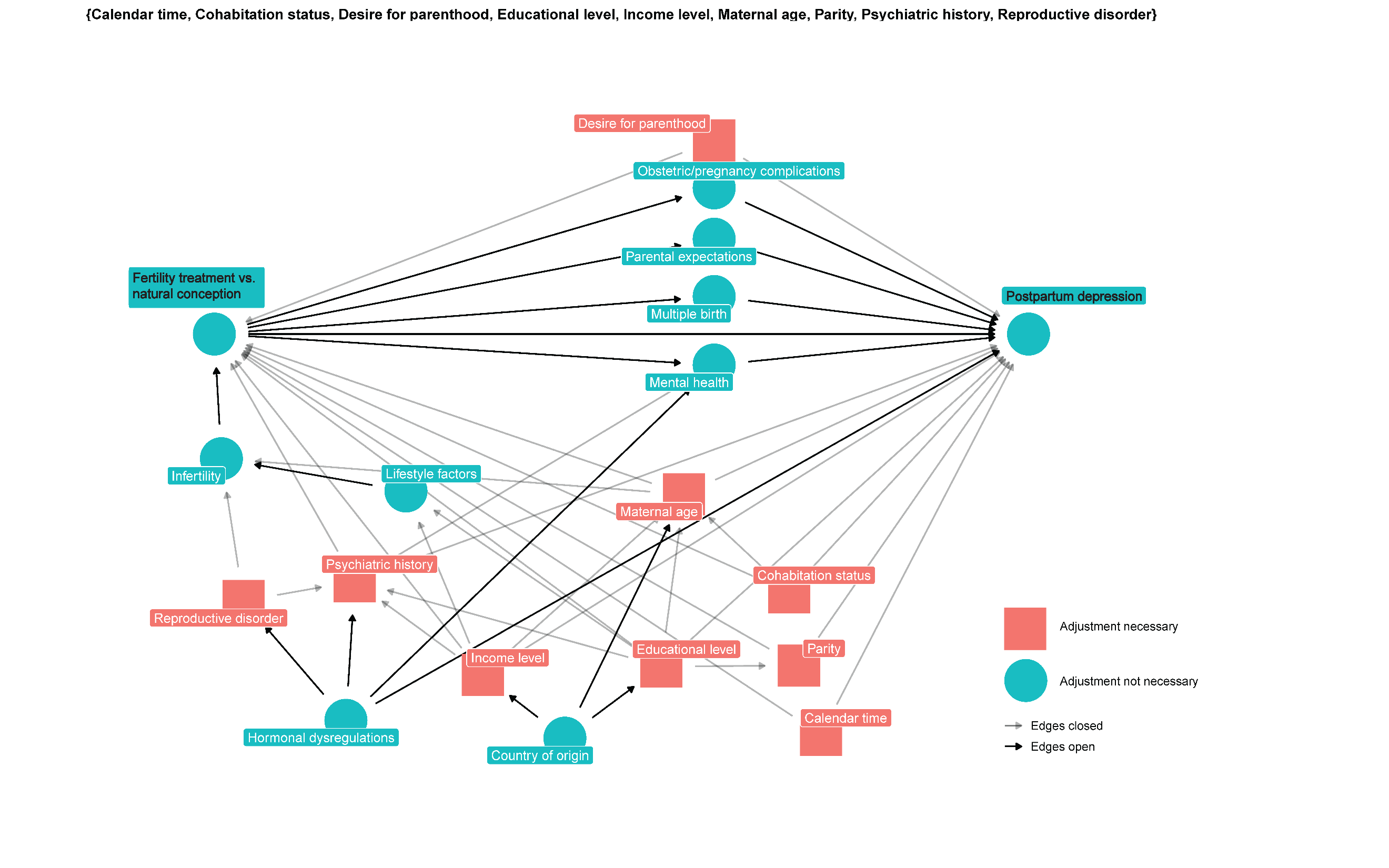
Figure S1: Directed acyclic graph on the association between MAR compared to spontaneous conception and risk of postpartum depression. The figure depicts variables necessary to adjust for to close open backdoor paths.**

**Figure S2: Directed acyclic graph for post hoc analysis in MAR only with exposures being a) type of MAR, b) duration of MAR treatment and 3) indication for MAR**

1. **
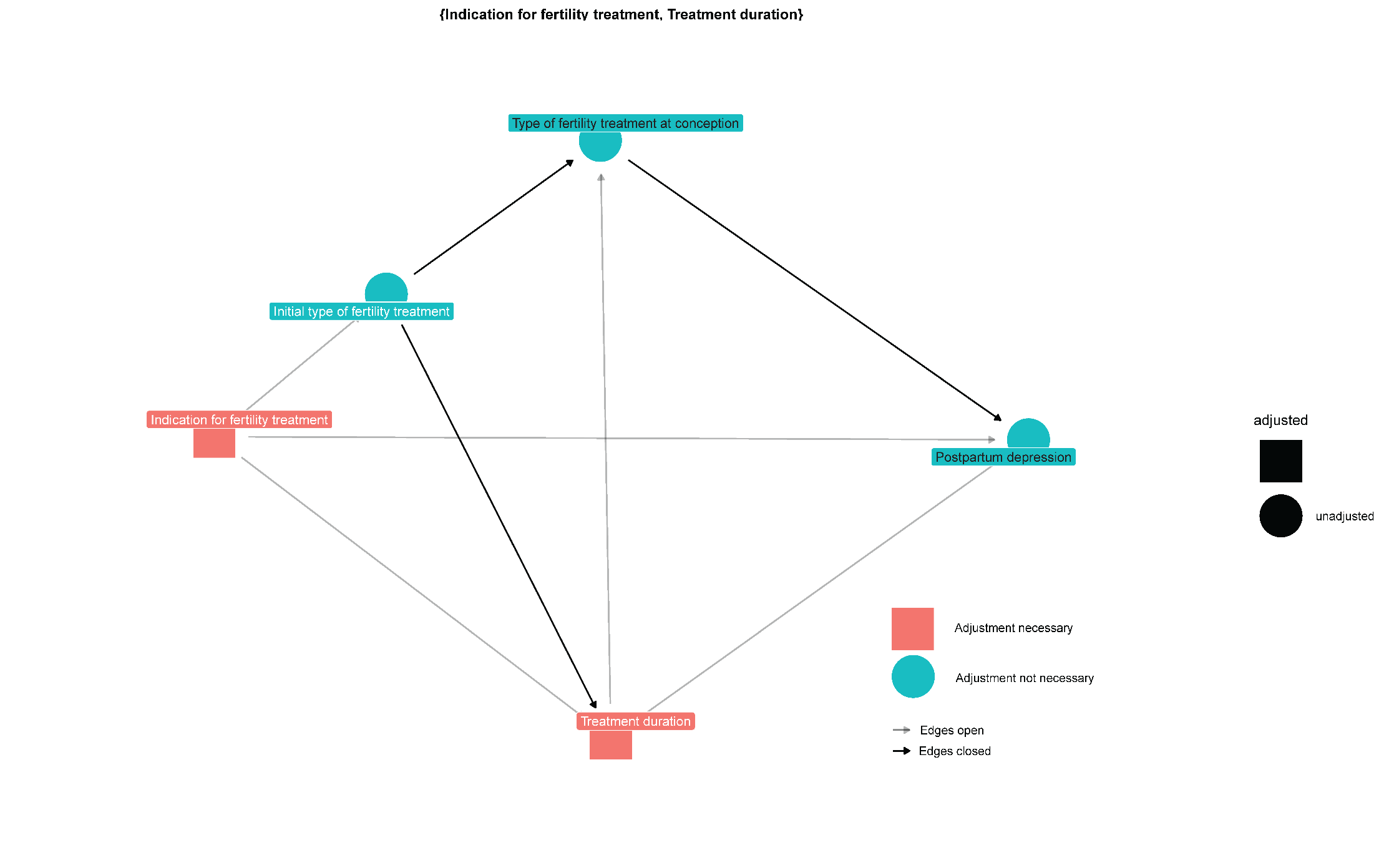
Type of MAR at conception as exposure**
2. **
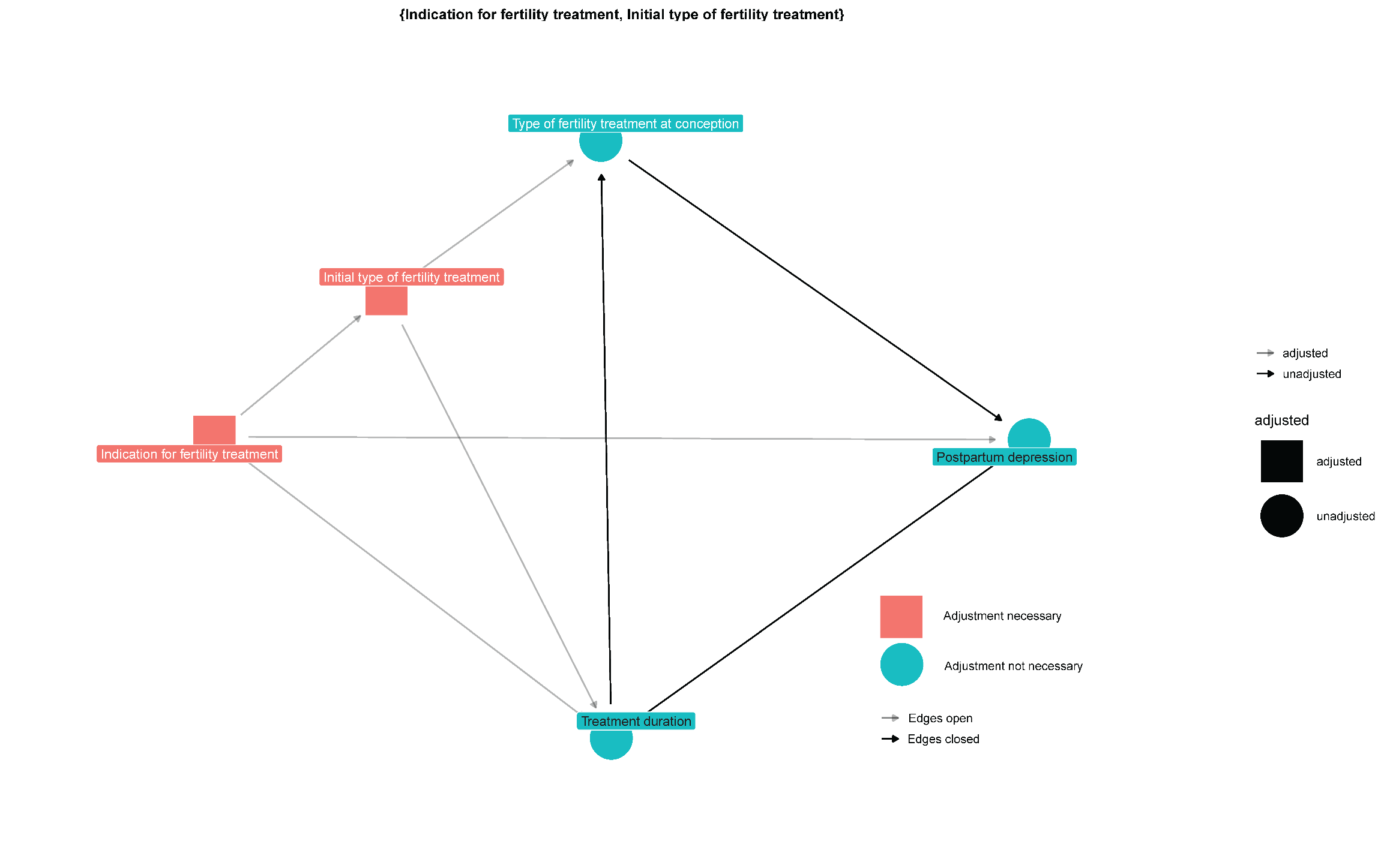
Treatment duration as exposure**
3. **Indication for MAR**

**
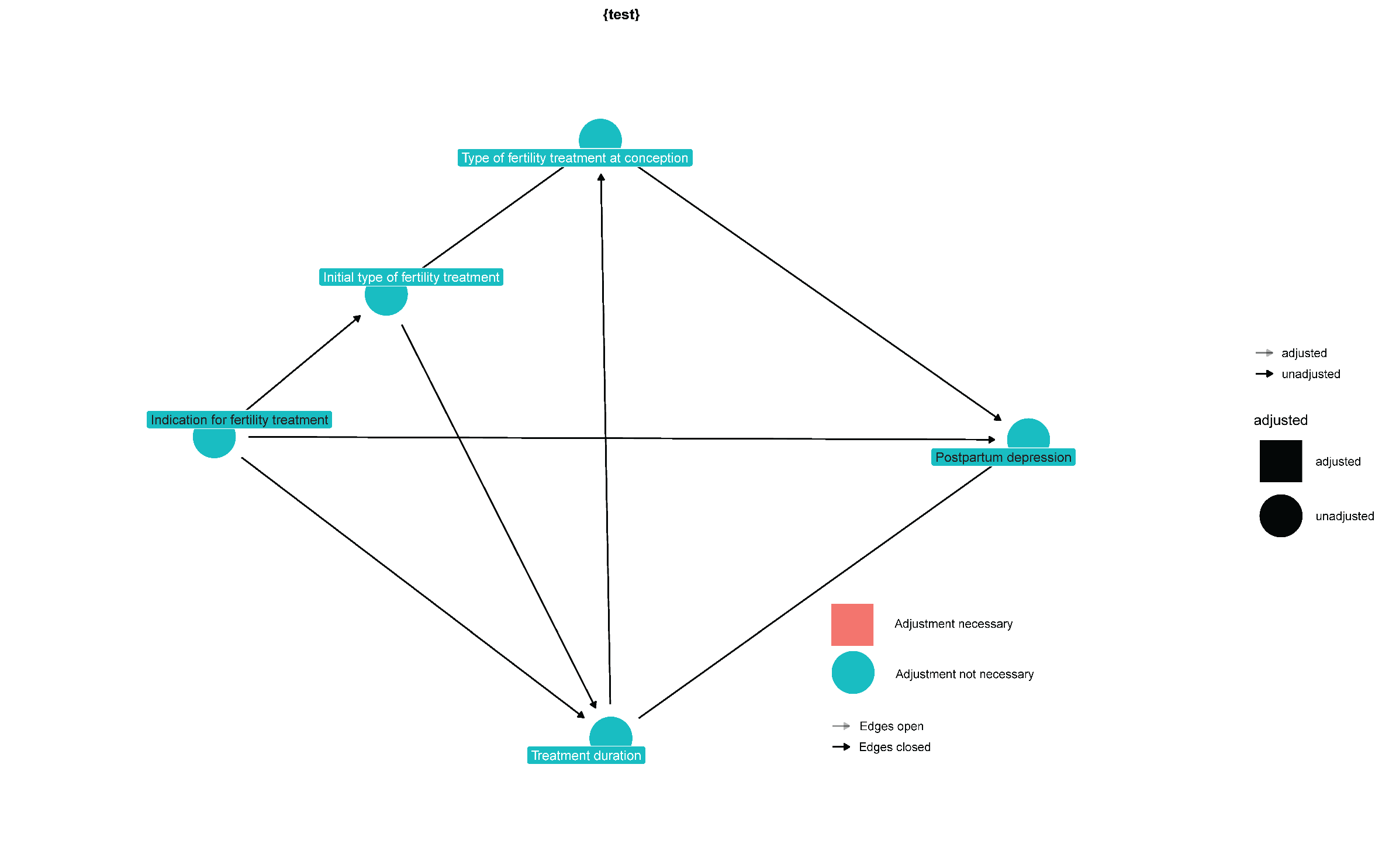
**

**Figure S3: Histogram of EPDS scores by exposure groups**

**
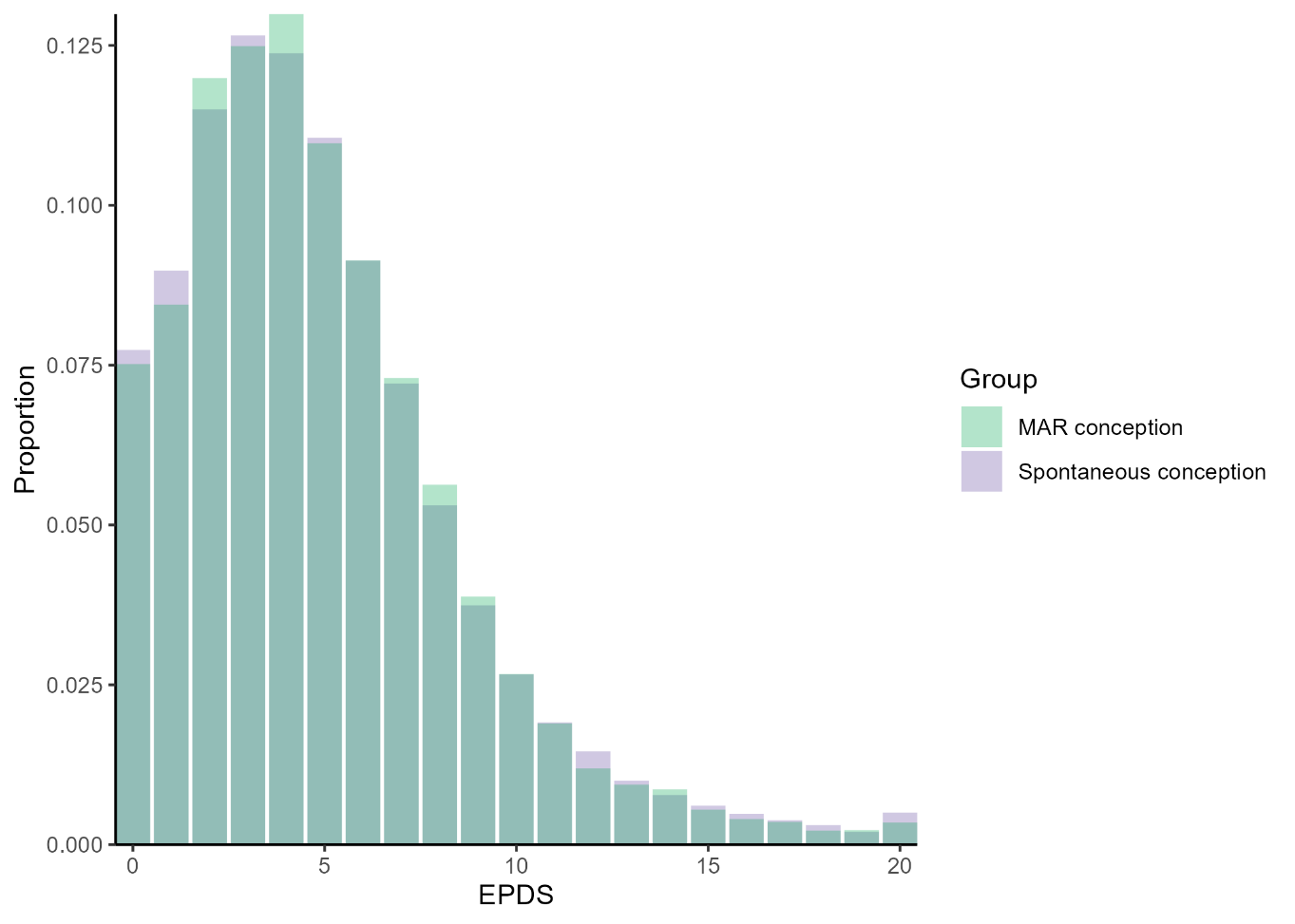
**

- *Note: Scores above 20 are collapsed to avoid too few observations in the data.*
